# Two common profiles exist for genomic oligonucleotide frequencies

**DOI:** 10.1186/1756-0500-5-639

**Published:** 2012-11-17

**Authors:** Shang-Hong Zhang, Lei Wang

**Affiliations:** 1 Key Laboratory of Gene Engineering of Ministry of Education, and Biotechnology Research Center, Sun Yat-sen University, Guangzhou, 510275, China

**Keywords:** Genomic sequence, Oligonucleotide, Frequency profile, GC content, Strand symmetry

## Abstract

**Background:**

It was reported that there is a majority profile for trinucleotide frequencies among genomes. And further study has revealed that two common profiles, rather than one majority profile, exist for genomic trinucleotide frequencies. However, the origins of the common/majority profile remain elusive. Moreover, it is not clear whether the features of common profile may be extended to oligonucleotides other than trinucleotides.

**Findings:**

We analyzed 571 prokaryotic genomes (chromosomes) and some selected eukaryotic nuclear genomes as well as other genetic systems to study their compositional features. We found that there are also two common profiles for genomic oligonucleotide frequencies: one is from low-GC content genomes, and the other is from high-GC content genomes. Furthermore, each common profile is highly correlated to the average profile of random sequences with corresponding GC content and generated according to first-order symmetry.

**Conclusions:**

The causes for the existence of two common profiles would mainly be GC content variations and strand symmetry of genomic sequences. Therefore, both GC content and strand symmetry would play important roles in genome evolution.

## Findings

Characteristics of oligonucleotide frequencies are basic features of genomic sequences. GC content and strand symmetry are two important ones among them. GC content (percentage of G + C to total number of nucleotides, varying from less than 20% to 75% among genomes) has been used in many analyses of the sequences of genes and genomes (for review see [1-3]). The other feature, the less well-known phenomenon of strand symmetry (the marked similarity of the frequencies of oligonucleotides to those of their respective reverse complements within single strands of sufficiently long genomic sequences, also called Chargaff’s second parity rule), exists in all cellular genomes studied (for review see [4-7]). Both GC content and strand symmetry, issues far from being conclusive, may provide clues to the profound understanding of genome evolution.

In addition to GC content variations among genomes and the ubiquitous phenomenon of strand symmetry, there are other important or interesting features of genomic oligonucleotide frequencies that deserve to be further studied. Examples may include: on one hand, the genomic signature, i.e., the set of all dinucleotide or higher-order oligonucleotide relative abundance values, which may be species-specific or taxon-specific [8,9]; on the other hand, the frequency conservation of some oligonucleotides across genomes, in which strand symmetry plays an important role [10]. Also, there exists the noticeable “majority profile” (referring to the average profile with which a large number of widely different cellular genomes may comply [11], and called a “common profile” in our study because a profile of this sort concerned generally less than half of the genomes analyzed; see also [12]) for genomic trinucleotide frequencies. It was reported that there is only one AT-rich majority profile for trinucleotide frequencies among genomes [11]. It was also proposed that the majority profile would be a reflection of general mechanisms of genome evolution [11] or a universal genome format that would result from numerous inversions/transpositions during genome evolution [13]. Yet, it was unclear why the majority profile is for low-GC content genomes rather than for high-GC content genomes. In this regard, further study with more genome samples has revealed that two common profiles, rather than only one majority profile, exist for trinucleotide frequencies of prokaryotic genomes [12] (one is similar to the majority profile reported previously [11], the other is GC rich). However, the causes and origins of the common or majority profile, features related to GC content and strand symmetry, remain elusive. Moreover, it is not apparent whether the features of common profile may be extended to oligonucleotides other than trinucleotides.

For the further understanding of the phenomenon of common profile, we analyzed the profiles of oligonucleotide frequencies of hundreds of prokaryotic genomes and some selected eukaryotic nuclear genomes as well as other genetic systems (organelle genomes, virus and phage genomes, and plasmids). We found that there are also two common profiles for genomic oligonucleotide frequencies (for each order of oligonucleotides): one is from low-GC content genomes, and the other is from high-GC content genomes. Furthermore, each of the two common profiles is highly correlated to the average profile of random sequences with corresponding GC content and generated according to first-order symmetry (strand symmetry for mononucleotides). Therefore, we conclude that the causes for the two common profiles (including the majority profile reported in [11]) would mainly be GC content variations and strand symmetry of genomic sequences.

## Materials and methods

### Whole-genome sequences analyzed

We downloaded the complete sequences of every species of archaea and bacteria that was available as of December 2008 from the NCBI ( http://ftp.ncbi.nih.gov/genomes/). For the species that have two or more strains or subspecies whose genomes have been sequenced, only one was taken randomly from each of them unless there is a difference of at least 1.0% of GC content between individual strains or subspecies of a species, in which case all the strains or subspecies were used in the analysis (see also Discussion). In total, 48 archaea and 509 bacteria were analyzed in the study, including genomes with GC content from 16.56% to 74.91%. For eukaryotic nuclear genomes, we analyzed particularly *Homo sapiens* and *Mus musculus* chromosomes (genomes with good isochore maps; sequences downloaded from UCSC Genome Bioinformatics: http://hgdownload.cse.ucsc.edu/goldenPath/), as well as the genomes with relatively high GC content: *Ashbya gossypii* (*Eremothecium gossypii*) ATCC 10895, *Encephalitozoon cuniculi* GB-M1, *Ostreococcus lucimarinus* CCE9901 and *Yarrowia lipolytica* CLIB122 (sequences from the NCBI). In addition, we selected representatively from the NCBI ( http://www.ncbi.nlm.nih.gov/entrez/query.fcgi?db=Genome) the complete sequences of 182 mitochondrial genomes, 170 chloroplast genomes (all available samples at the time of our study), 96 plasmids, 64 viruses and 64 phages. The list of all analyzed genomes and sequences is available in Additional file 1.

### Analysis of oligonucleotide frequency profiles

We calculated the frequency (percentage) of every mono-, di-, tri-, tetra-, penta- and hexanucleotide in each prokaryotic genome, and also that of every trinucleotide in each of the nuclear genomes (chromosomes), organelle genomes, virus and phage genomes, and plasmids studied. Overlapping oligonucleotides were counted in the calculations (see also [6,10]). We analyzed the individual chromosomes in multi-chromosomed prokaryotic genomes separately when there is a difference of at least 1.0% of GC content between chromosomes (see also Discussion). As there are considerable intra-genomic variations of GC content (isochores) in nuclear genomes, we analyzed their GC content and trinucleotide frequencies segment by segment (using a non-overlapping moving window of 100 kb; see also [11,14,15]).

The frequency values of all oligonucleotides of an order obtained from a genome (sequence) and arranged in a determined way constitute the frequency profile of oligonucleotides of that order for the genome (sequence). Oligonucleotide frequency profiles were analyzed in principle in the same way as described in [11]. We evaluated the relationship of any two profiles of the same order by calculating the Pearson correlation coefficient (*r*). Our test has shown that “profile correlation” corresponds to “profile similarity” or “profile difference” to a reasonable extent, except that the correspondence is not sensitive for mononucleotide frequency profiles^a^. In the correlation analysis, the significance (*P*-value) of *r* was also taken into account. Both strands of a genome or of a sequence were considered (only the more appropriate one was chosen) when evaluating the relationship of its profile to others (see also [6]; little difference between the profile of a strand and that of its complementary strand if there is excellent strand symmetry). We employed the cluster analysis (more specifically, unweighted pair group method with arithmetic mean, UPGMA) for the classification of profiles according to their mutual correlation coefficients. Two parameters, *c*_div_ and SD, were used to illustrate the overall similarity of profiles clustered in a group by UPGMA. The parameter *c*_div_ denotes class diversity, whose value is 1 − average (*c*_*XY*_), where *c*_*XY*_ represents all the correlation coefficients calculated between any two profiles in the group; SD is the standard deviation of the correlation coefficients calculated between any two profiles in the group (see [11] for more detail). Under the criteria *c*_div_ < 0.10 and SD < 0.06, a group clustered by UPGMA is considered as a class (the criteria would be appropriate in most cases for grouping similar profiles together; for higher-order oligonucleotides or for virus and phage genomes, the criteria may be less strict; see also Discussion). Genomes are classified into the common class (with numerous members in a class), the minority class (with much fewer members in a class than the common class), and the violator class (with members devoid of the feature of strand symmetry in a class) (see also [11,12]). The average profile of all profiles of the members of a common class is called a common profile. Minority profiles and violator profiles were produced in similar ways. The level of strand symmetry was measured by *c*_WC_ (degree of compliance, defined as the correlation coefficient between the profiles of the two strands of a genome or sequence) (see also [6,11]).

Random sequences of 8 Mb were generated according to first-order symmetry, with GC content varying from 16% to 75% at 1% intervals. In total, 300 random sequences were obtained (five sequences were generated around each value of GC content). Trinucleotide frequency profiles of these random sequences were analyzed as prokaryotic genomes. This analysis was to figure out how the variations of GC content would influence the frequency profiles, and to determine whether random sequences somewhat independent of natural sequences could also have similar traits as the common profiles.

The calculations of oligonucleotide frequencies and the generation of random sequences were performed with computer programs written in Perl.

## Results

### Two common profiles for oligonucleotide frequencies of prokaryotic genomes

Regardless of orders of oligonucleotides, the profiles of a large number of AT-rich prokaryotic genomes (chromosomes) studied may be grouped together with a small *c*_div_ and SD. Therefore, there is a low-GC content common class (common class 1) for each order of oligonucleotides (Table 1). The same is true of GC-rich prokaryotic genomes (chromosomes) studied, and there is also a high-GC content common class (common class 2) for each order of oligonucleotides (Table 1). Note that profiles of genomes with GC content around 50% could not be grouped together with a small *c*_div_ and SD. These genomes belong to the minority class, and there is not an intermediate-GC content common class (see also Additional file 2 and Additional file 3).

**Table 1:** Characteristics of oligonucleotide frequency profiles of common class 1 and common class 2 prokaryotic genomes

**Oligonucleotide**	**Common class 1**				**Common class 2**			
	**Member**	**Range (%)**	***c***_**div**_	**SD**		**Member**	**Range (%)**	***c***_**div**_	**SD**
Mononucleotide	300	16.56–49.72	0.006	0.020	264	50.32–74.91	0.004	0.015
Dinucleotide	218	16.56–46.08	0.058	0.048	183	54.60–74.91	0.070	0.052
Trinucleotide	184	16.56–42.83	0.075	0.045	160	58.02–74.91	0.086	0.051
Tetranucleotide	174	16.56–43.13	0.097	0.054	161	58.02–74.91	0.124	0.117
Pentanucleotide	203	16.56–43.87	0.102	0.062	139	57.23–74.91	0.087	0.051
Hexanucleotide	158	16.56–42.68	0.143	0.073	155	58.02–74.91	0.160	0.076

Member: number of members; Range: range of GC content; *c*_div_: class diversity; SD: standard deviation.

It is clear that there is a tendency for the number of members of common class 1 or of common class 2 to decrease as the order of oligonucleotides increases (Table 1). This would be due to the situation that the number of different oligonucleotides in each frequency profile and the diversity of the profiles themselves increase with the order of oligonucleotides, which may considerably influence the values of the mutual correlation coefficients. In fact, with only four frequency values, the profiles of mononucleotides exhibit similar shapes (tendencies of variations) for almost all genomes with GC content lower than 50% as well as for those with GC content higher than 50%. Therefore, the two parameters measuring the overall similarity of profiles (*c*_div_ and SD, especially *c*_div_) are very small in these situations. Beginning with dinucleotides, the number of frequency values in each profile dramatically increases, and the shapes of profiles are more and more diverse with the increase of order of oligonucleotides. Also, it seems that the increase of diversity is more obvious for common class 2 (Table 1). The difference between the number of members of common class 1 and that of common class 2 is mainly due to the difference of number of samples between low-GC and high-GC content genomes (see Additional file 1).

**Figure 1:**
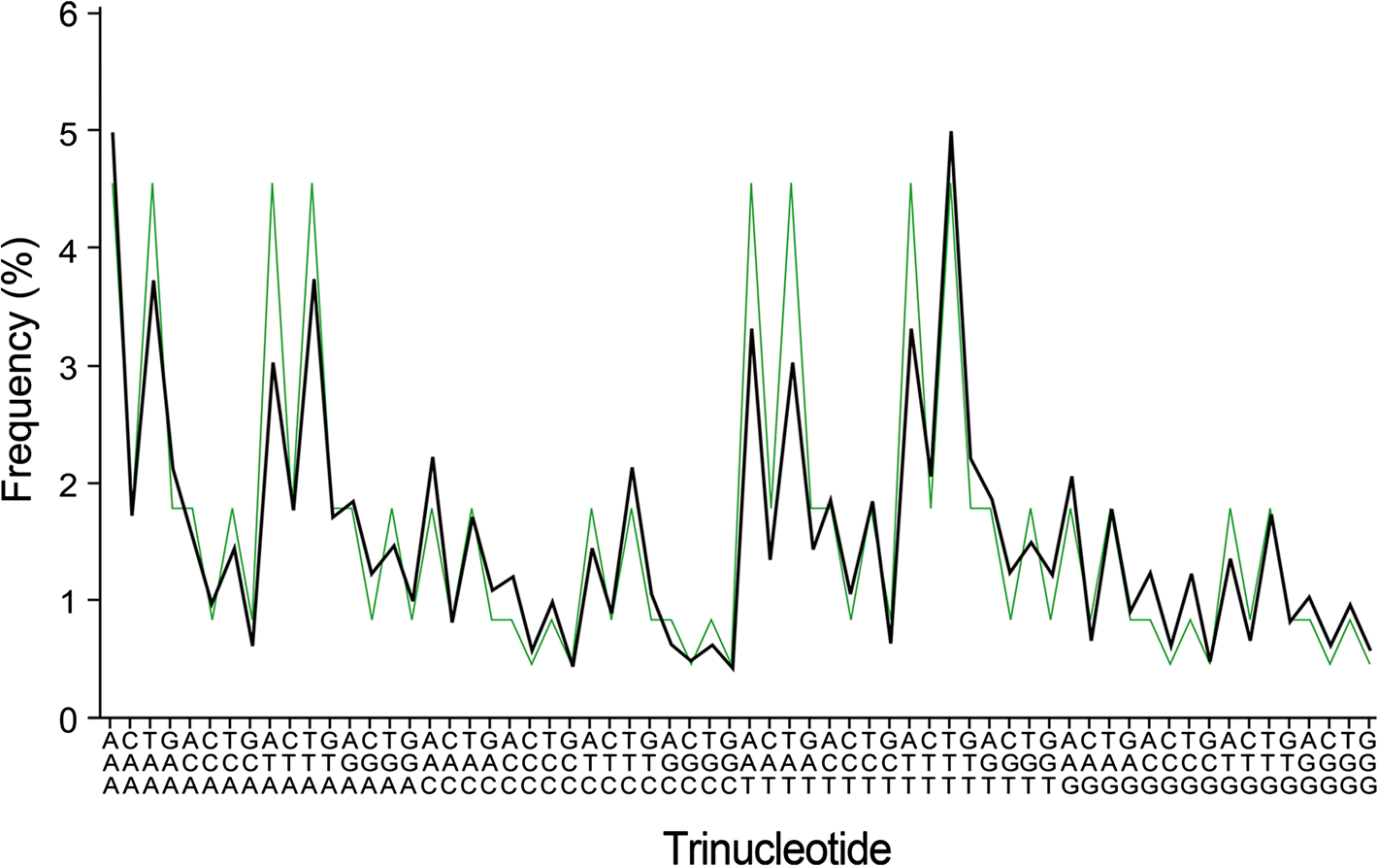
**Common profile 1 for genomic trinucleotide frequencies and the average profile of its corresponding random sequences.** Common profile 1 (black line) was obtained as the average of similar trinucleotide frequency profiles of 184 prokaryotic genomes (chromosomes) with GC content ranging from 16.56% to 42.83%. Alongside common profile 1 is the average trinucleotide frequency profile (green line) of 140 random sequences with GC content of 16%–43% and generated according to first-order symmetry. Trinucleotides in abscissa should be read vertically from bottom to top.

**Figure 2:**
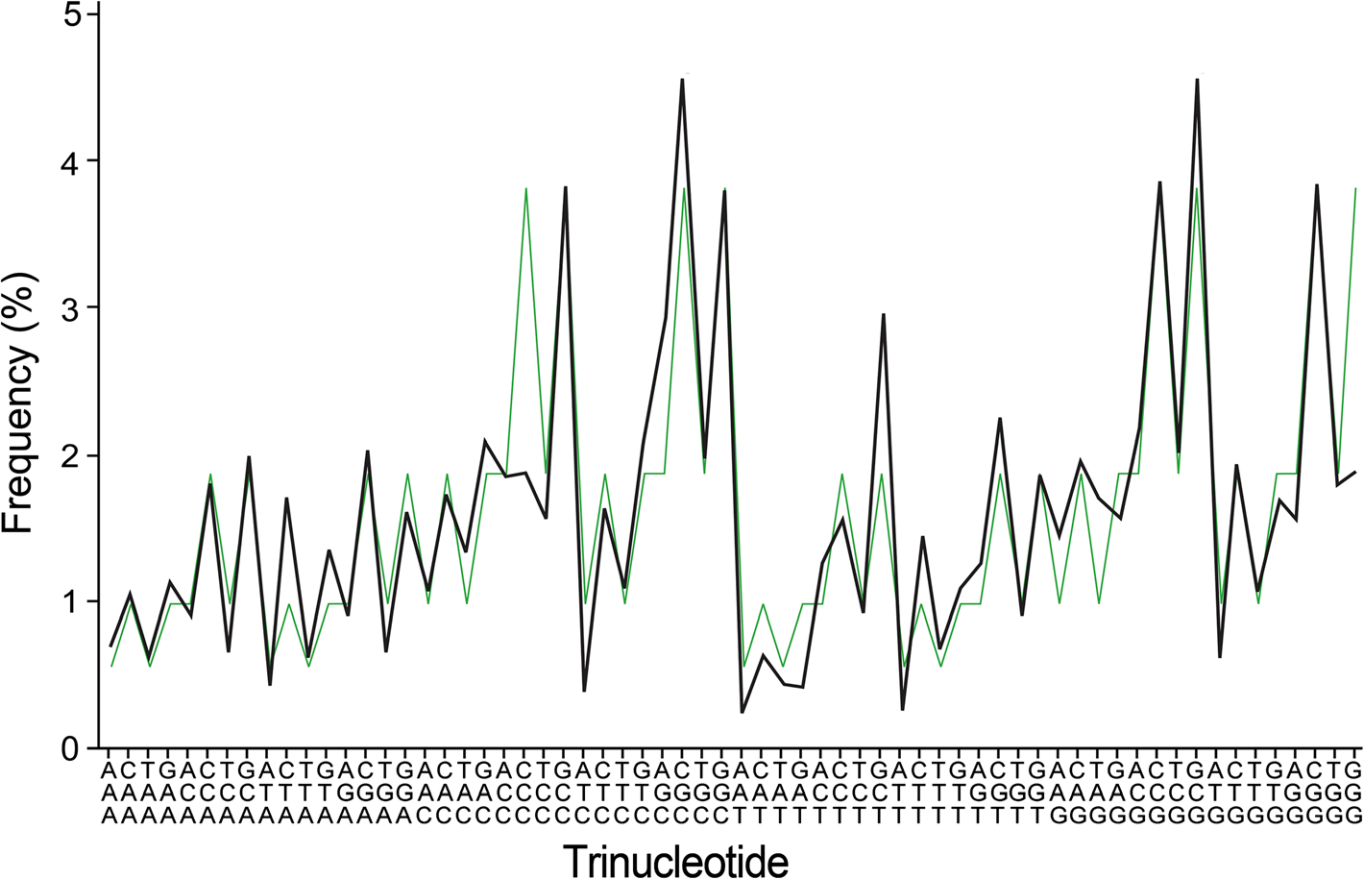
**Common profile 2 for genomic trinucleotide frequencies and the average profile of its corresponding random sequences.** Common profile 2 was obtained as the average of similar trinucleotide frequency profiles of 160 prokaryotic genomes (chromosomes) with GC content ranging from 58.02% to 74.91%. Alongside common profile 2 is the average trinucleotide frequency profile of 90 random sequences with GC content of 58%–75% and generated according to first-order symmetry. For other explanations see Figure 1.

For each order of oligonucleotides, the average profiles of common class 1 and common class 2, respectively, would correspond to a low-GC content common profile (common profile 1) and a high-GC content common profile (common profile 2). An example of the common profiles for trinucleotide frequencies is shown in Figure 1 and Figure 2 (see also [12] and Additional file 3).

In addition to the two common profiles, there exist the minority profiles for genomes of the minority class (most of them with intermediate-GC content). However, the number of genomes complying with a specific minority profile is relatively small. Also, these genomes are with similar GC content, and a high proportion of them would be closely related in terms of phylogeny (see Additional file 3).

**Figure 3:**
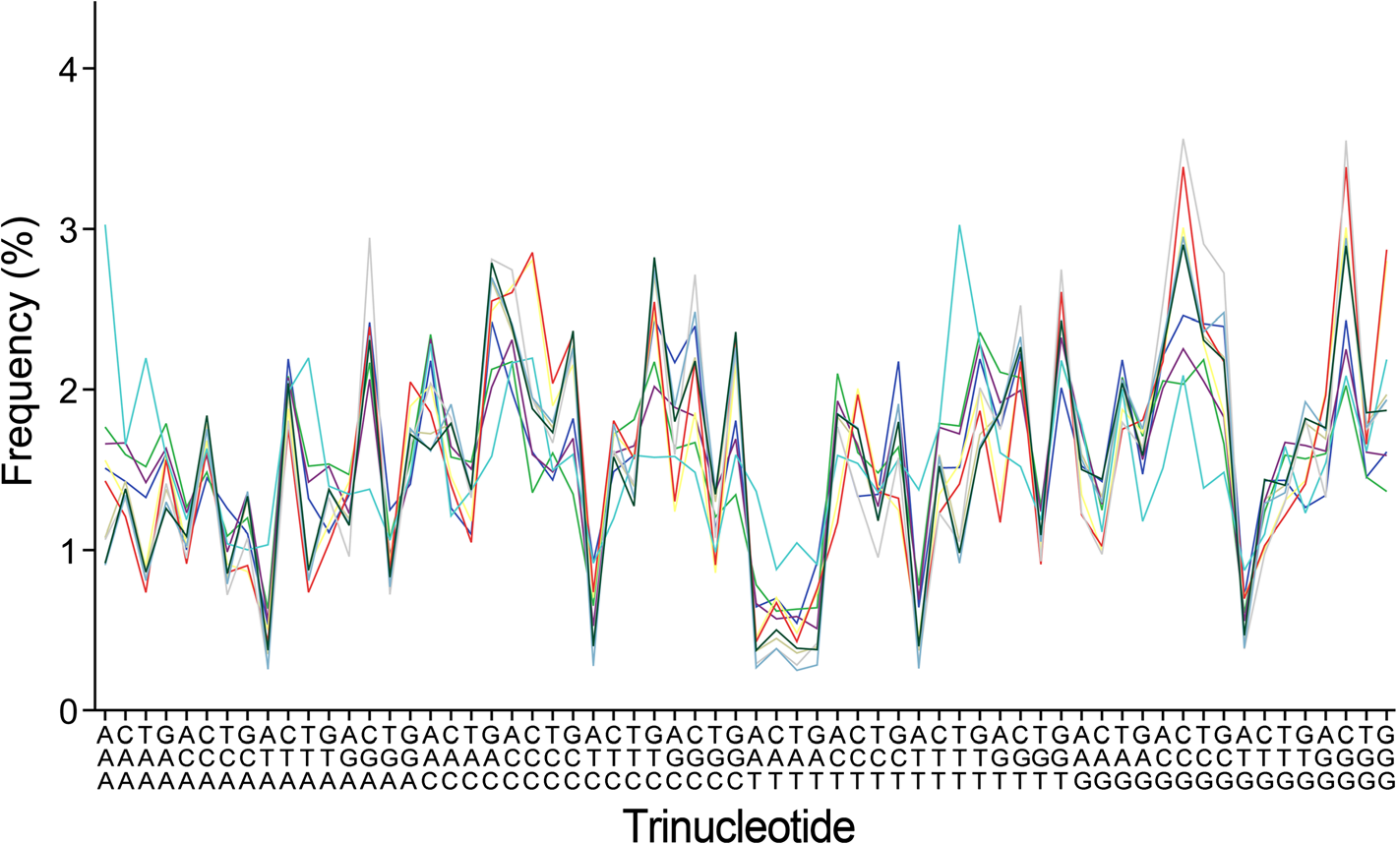
**Trinucleotide frequency profiles of the genomes of species from the genus *Synechococcus.*** Their genomic GC content varies from 49.63% to 60.84%. The profiles differ considerably from one another (10 members; *c*_div_ = 0.215, SD = 0.190). None of the profiles complies well with either common profile 1 or common profile 2. For the explanation of abscissa see Figure 1.

Although closely related genomes may have similar profiles, it is apparent that the profile of a prokaryotic genome would mainly depend on its GC content rather than its phylogenetic position. Most, if not all, At-rich genomes are members of common class 1, regardless of their phylogenetic positions; the same situation exists for GC-rich genomes as members of common class 2 (Additional file 2, see also [12]). In addition, oligonucleotide frequency profiles of genomes of the same genus with widely different GC content are considerably different from one another (see Figure 3 for trinucleotide frequency profiles). Indeed, even for the strains or subspecies of a species, the genomic oligonucleotide frequency profiles may be very different if they are with considerably different GC content. An exceptional example may be found for the species *Prochlorococcus marinus*: among the 12 strains studied, 10 strains (GC content 30.79%–38.01%) are members of common class 1 in terms of oligonucleotide frequency profiles, while neither of the other two strains (GC content 50.01% and 50.74%, respectively) could have an oligonucleotide frequency profile complying with either common profile (Additional file 2). On the other hand, it is worthwhile to note that, under our criteria, a high proportion of archaeal genomes that are well within the GC content range of common class 1 or of common class 2 are not members of the common class, especially in terms of tri-, tetra- and hexanucleotide frequency profiles and for genomes within the GC content range of common class 2 (Additional file 1 and Additional file 2; yet their profiles are not very different from the corresponding common profile, data not shown).

Therefore, all the above results indicate that there are also two common profiles for genomic oligonucleotide frequencies: one for low-GC content genomes, the other for high-GC content genomes. For intermediate-GC content genomes, on the other hand, no common profile of their own exists although some of them may be members of common class 1 or of common class 2 (especially in terms of mono- and dinucleotide frequency profiles; see also Additional file 2).

### Profiles of trinucleotide frequencies of random sequences generated according to first-order symmetry

To study further the origins of the common profiles, we analyzed the trinucleotide frequency profiles of random sequences. Random sequences with an equal proportion of each nucleotide could hardly have a profile close to those of natural genomes [11]. Therefore, random sequences possessing some general characteristics of natural genomes should be used instead. There is a wide range of GC content in natural genomes, and the above results show that GC content is very important in determining an oligonucleotide frequency profile. In addition, strand symmetry is a ubiquitous phenomenon of prokaryotic and nuclear genomes. Considering all these features, we analyzed long random sequences with GC content varying as that of natural genomes and with some traits of strand symmetry (random sequences generated according to first-order symmetry and with different GC content). For oligonucleotides of the same order in these sequences, those of the same GC content will be with practically the same frequencies (see also Figure 1 and Figure 2).

Trinucleotide frequency profiles of the random sequences with similar GC content are generally very closely related. Nevertheless, there are also two large groups revealed by cluster analysis in accordance with their GC content (lower than 50% versus higher than 50%; Additional file 4). Among the 300 random sequences generated, 170 ones are in group 1 (GC content 16%–49%; *c*_div_ = 0.034, SD = 0.039), while 125 ones are in group 2 (GC content 51%–75%; *c*_div_ = 0.020, SD = 0.023). The corresponding average profiles (average profile 1^ran170^ and average profile 2^ran125^) for these two groups of random sequences are highly correlated with common profiles 1 and 2 of prokaryotic genomes, respectively (*r*_1-1_ = 0.938, *P* < 0.0001; *r*_2-2_ = 0.879, *P* < 0.0001). In spite of the very high average correlation, individual trinucleotide frequency profiles of the random sequences with GC content close to 50% comply less well with the corresponding common profile (Additional file 4). When only the random sequences with GC content ranging from 16% to 43% (corresponding to the range for common profile 1; 140 sequences) and from 58% to 75% (corresponding to the range for common profile 2; 90 sequences) are taken into account, it produces two average profiles (average profile 1^ran140^, *c*_div_ = 0.023, SD = 0.026; average profile 2^ran90^, *c*_div_ = 0.010, SD = 0.012) that correlate also very well with common profiles 1 and 2 of prokaryotic genomes, respectively (*r*_1'-1_ = 0.938, *P* < 0.0001; *r*_2'-2_ = 0.877, *P* < 0.0001; Figure 1 and Figure 2). Moreover, each trinucleotide frequency profile of the random sequences for average profile 1^ran140^ or average profile 2^ran90^ complies very well with the corresponding common profile 1 or common profile 2 (Additional file 4). Overall, the correlation between common profile 1 and its corresponding random sequences is better than that between common profile 2 and its corresponding random sequences (note that the CCC and GGG peaks are not obvious for common profile 2 as compared with those of its corresponding random sequences; Figure 2).

It is noteworthy that, according to the cluster analysis, there is no grouping of random sequences of intermediate-GC content. This would also be an indication that no intermediate-GC content common profile could exist among natural genomes.

These results would imply that GC content as well as strand symmetry would be the key factors for oligonucleotide frequency profiles of natural genomes.

### Profiles of trinucleotide frequencies of eukaryotic genomes, organelle, virus and phage genomes, and plasmids

Compared with prokaryotic genomes, the GC content of eukaryotic nuclear genomes (and the isochores within) and organelle genomes is relatively low. On the other hand, virus and phage genomes as well as plasmids may be with similar GC content variations as prokaryotic genomes (see also Additional file 1). Moreover, the phenomenon of strand symmetry is less obvious or even absent in plasmids and in organelle, virus and phage genomes [6,10,16,17]. Therefore, it is interesting to analyze these genomes or sequences to see whether common profiles also exist.

It has been shown that some large segments of nuclear genomes may be grouped with certain prokaryotic genomes to produce a low-GC content majority profile for trinucleotide frequencies [11]. Our results also indicate that trinucleotide frequency profiles of many large segments (with relatively uniform GC content throughout a segment) of the human and mouse chromosomes comply well with common profile 1 of prokaryotic genomes (*r* ≥ 0.9, *P* < 0.0001; Additional file 5). Examples may include: human chromosome 1, positions 68,600,001–83,800,000 (15.2 Mb); human chromosome X, positions 77,400,001–99,100,000 (21.7 Mb); mouse chromosome 14, positions 78,300,001–89,800,000 (11.5 Mb). In fact, the trinucleotide frequency profile of an entire chromosome, such as human chromosome 3, 4, 5, 6, 13, 18 or X, may comply well with common profile 1 (*r* > 0.85, *P* < 0.0001; Additional file 5). However, none of the segments larger than or equal to 100 kb of the human and mouse chromosomes, as well as of the chromosomes of *Encephalitozoon cuniculi* and *Yarrowia lipolytica*, could have a trinucleotide frequency profile close to common profile 2 of prokaryotic genomes. On the other hand, the chromosomes of *Ashbya gossypii* (*Eremothecium gossypii*) and *Ostreococcus lucimarinus*, the two eukaryotes with the highest genomic GC content in our study (51.94% and 60.44%, respectively), may contain large segments that could have a trinucleotide frequency profile close to common profile 2 (*r* > 0.8, *P* < 0.0001; Additional file 5). Our results and the previous data [11] taken together, it is clear that most nuclear genomes may contain many large segments or isochores whose trinucleotide frequency profiles comply well with the low-GC content common profile; nevertheless, only the nuclear genomes with the highest GC content could have large segments whose trinucleotide frequency profiles comply well with the high-GC content common profile.

As for organelle genomes, there is a common profile for trinucleotide frequencies of mitochondrial genomes; there is also an overall common profile for chloroplast genomes (actually almost all of them conform well to this common profile). The common class of mitochondrial genomes in our analysis contains 73 members (GC content 14.01%–34.42%; *c*_div_ = 0.072, SD = 0.043) that produce an average profile highly correlated to common profile 1 (*r* = 0.952, *P* < 0.0001; Additional file 6, see also [11]). It is not unusual to have this result because the levels of strand symmetry of the members of this common class are all considerably high. The mitochondrial genomes without the feature of strand symmetry are in the violator class (see also Additional file 6 and [11]). The trinucleotide frequency profiles of all chloroplast genomes studied, except the three with the highest GC content (42.14%, 44.02% and 51.00%, respectively), may be grouped into two subclasses (Additional file 6). The first subclass contains 112 members (GC content 32.90%–42.01%; *c*_div_ = 0.019, SD = 0.014); the second subclass contains 55 members (GC content 19.48%–40.47%; *c*_div_ = 0.075, SD = 0.050). The average profiles of each of the two subclass are highly correlated (*r* = 0.921, *P* < 0.0001); they are both, with all the members exhibiting well the feature of strand symmetry, highly correlated to common profile 1 (with *r* as high as 0.937 and 0.988, respectively, *P* < 0.0001; Additional file 6, see also [11]). Therefore, these two subclasses may be grouped into an overall (a little less strict) common class (*c*_div_ = 0.069, SD = 0.061). Also, the two subclasses together produce an average profile highly correlated to common profile 1 (*r* = 0.977, *P* < 0.0001; Additional file 6). It is clear that both mitochondrial and chloroplast genomes have only one common profile (the low-GC content one) because their genomic GC content rarely exceeds 50%.

With similar GC content variations as prokaryotic genomes, plasmids may have two common profiles of their own for trinucleotide frequencies (Additional file 6). The first one in our study would be the average profile of 26 members (GC content 23.10%–43.61%; *c*_div_ = 0.090, SD = 0.051), which is highly correlated to common profile 1 (*r* = 0.992, *P* < 0.0001). The second one was averaged from 17 members (GC content 54.11%–62.33%; *c*_div_ = 0.080, SD = 0.046); it is highly correlated to common profile 2 (*r* = 0.967, *P* < 0.0001). All the members for these two profiles are with considerably high trinucleotide symmetry levels (Additional file 6).

On the other hand, it seems that virus and phage genomes are more diverse in trinucleotide frequency profiles, and the common class is not apparent. This would be due to the facts that: (1) most of them are with GC content lower than 50%, hence no sufficient samples for a high-GC content common class in our study (Additional file 1); and (2) some low- or high-GC content samples are with low symmetry levels. In spite of this situation, in both virus and phage genomes there exist ten or so low-GC content members whose profiles comply quite well with common profile 1; there are also several high-GC content members whose profiles comply quite well with common profile 2 (Additional file 6). Interestingly, these members are as well with a relatively high symmetry level for trinucleotide frequencies.

Overall, the results from eukaryotic genomes, organelle, virus and phage genomes, and plasmids confirm our conclusion from the analysis of prokaryotic genomes and random sequences.

## Discussion

GC content is an important factor for the oligonucleotide frequency profile of a genome. Therefore, we included in the analysis samples all the strains or subspecies of a prokaryotic species between which there is a difference of at least 1.0% of GC content. For the same reason, we analyzed the individual chromosomes in multi-chromosomed prokaryotic genomes separately when there is a difference of at least 1.0% of GC content between chromosomes. On the other hand, as the GC content variations among strains or subspecies of a species are generally small, our sampling strategy provided equivalent results to those of “one species–one genome” strategy (data not shown, the exceptional situation of *Prochlorococcus marinus* would not change considerably the concerned common profile). The same is true for the analysis of individual chromosomes separately or together in multi-chromosomed prokaryotic genomes (data not shown).

There may be intrinsic correlations within an oligonucleotide frequency profile (e.g., the frequency value of an oligonucleotide and that of its reverse complement are very similar in long genomic sequences). In our case, however, intrinsic correlations within a profile would not prevent from applying correlation analysis to comparing two profiles (data not shown).

There is a common profile for low-GC content genomes, and another for high-GC content genomes. The high-GC content common profile seems to be more specific to natural genomic sequences, for: (1) it would correlate less well with the corresponding random sequences compared with the low-GC content common profile; and (2) the individual profiles that produce the common profile would be a little more diverse (see Table 1). On the other hand, regardless of the existence of low-GC content and high-GC content common frequency profiles for each order of oligonucleotides, no common profile could be found for genomes with GC content around 50%. This is because the variations of frequencies of oligonucleotides of the same order are limited in these genomes, and there is hardly a consensus for the tendencies of variations. Therefore, no common profile for these genomes exists according to the correlation analysis. Even for random sequences generated according to first-order symmetry, those with GC content of 50% could hardly be grouped with others in cluster analysis. And this is also the reason why the trinucleotide frequency profiles of natural genomes differ substantially from those of random sequences with %A = %T = %C = %G (GC content = 50%), in addition to the fact that these random sequences could not comply with the majority profile (see [6,11]). Alternatively, random sequences generated according to first-order symmetry and with GC content in the range for a common profile conform well to the profile.

The size of a class of profiles (the number of members) would be influenced by the criteria of grouping, i.e., the limits of *c*_div_ and SD. However, providing the values of *c*_div_ and SD are within a reasonable range, common class 1 and common class 2 emerge, and the overall common profiles would not change considerably with the size of the common class (in our analysis, oligonucleotide frequency profiles were classified by UPGMA at early stages into two large groups and a number of small groups). As mononucleotide frequency profiles are not sensitive in terms of *c*_div_, almost all genomes with GC content lower than 50% are classified into common class 1, and almost all with GC content higher than 50% into common class 2. The size of the common class may be reduced by applying a much stricter criterion. On the other hand, the two mononucleotide common profiles would not change considerably either.

Our results show that two common profiles exist for genomic oligonucleotide frequencies (at least for mononucleotides through hexanucleotides). Besides appropriate genomic GC content, strand symmetry is very important for the appearance of a common frequency profile. GC content plays an important role in determining the basic pattern of a profile, while strand symmetry confines the pattern of variation of a profile. In principle, appropriate GC content and strand symmetry are sufficient to render a genome to be a member of a common class. Genomes without either sufficiently low (high) GC content or a sufficiently high level of strand symmetry could not produce a common profile. Of course, genomes with appropriate GC content and strand symmetry could not all in the common class.

Therefore, the causes for the existence of two common profiles (including the majority profile reported previously [11]) would mainly be GC content variations and strand symmetry of genomic sequences. In other words, GC content and strand symmetry are primary; the common profiles would be secondary issues to these two primary features (see also [12]), which would underline the importance of the phenomena of GC content variations among genomes and strand symmetry in the study of genome evolution. It has been proposed that strand symmetry would be a very primitive trait of genomic sequences [7,10]. If that is the case, the emergence of two common profiles would largely depend on the origins of GC content variations among genomes (see also [12]).

### Availability of supporting data

The data sets supporting the results of this article are included within the article and its additional files.

## Endnotes

^a^ The issue about “profile correlation” and “profile similarity” may be illustrated by the following examples:

Consider profile 1 (A = 0.1, C = 0.4, T = 0.1, G = 0.4) and profile 2 (A = 0.2, C = 0.3, T = 0.2, G = 0.3). The correlation coefficient between profile 1 and profile 2 is 1. Then, consider profile 1 and another profile (profile 3 with A = 0.15, C = 0.35, T = 0.15, G = 0.35). They are apparently more similar (or less different) than profile 1 and profile 2 (Additional file 7). Yet, the correlation coefficient between profile 1 and profile 3 is also 1. The reason for the equality of the correlation coefficients is that there are only two effective points in the scatter plot for any two of these profiles. For example, the two effective points are (0.1, 0.2) and (0.4, 0.3) in the scatter plot for profile 1 and profile 2. According to these results, it seems that the profile correlation does not correspond to the profile similarity (or the profile difference).

However, the situations are different when it turns to dinucleotide and higher-order oligonucleotide frequency profiles. Take also the data of mononucleotide profiles 1, 2 and 3. We referred to their corresponding dinucleotide frequency profiles (dinucleotide frequency values purely determined by the corresponding mononucleotide frequencies) as profile 1', profile 2' and profile 3', respectively. The comparison of profile 1' and profile 2', and that of profile 1' and profile 3' are shown in Additional file 7. Profile 1' and profile 3' are more similar than profile 1' and profile 2'. The correlation coefficient between profile 1' and profile 3' is 0.992, and that between profile 1' and profile 2' is 0.966. There is correspondence between the profile correlation and the profile similarity in this case. The same is true of the corresponding trinucleotide frequency profiles.

In fact, even for mononucleotide frequency profiles of natural sequences, the correspondence between profile correlation and profile similarity also exists, although not as apparent as dinucleotide and higher-order oligonucleotide frequency profiles. This is because in natural genomes (sequences), the frequencies of A and T are rarely identical, so are the frequencies of C and G. If we change profile 1 above to A = 0.11, C = 0.39, T = 0.10, G = 0.40 (profile 1^v^), profile 2 to A = 0.20, C = 0.30, T = 0.21, G = 0.29 (profile 2^v^), and profile 3 to A = 0.15, C = 0.35, T = 0.16, G = 0.34 (profile 3^v^), then the correlation coefficient between profile 1^v^ and profile 3^v^ is 0.996, and that between profile 1^v^ and profile 2^v^ is 0.989. The correlation coefficients are usually high between profiles with similar trend. The difference of profile correlation is small, but does exist and correspond to profile similarity (or profile difference).

Therefore, we may conclude that “profile correlation” corresponds to “profile similarity” to a reasonable extent, except that the correspondence is not sensitive for mononucleotide frequency profiles.

## Abbreviation

UPGMA: Unweighted pair group method with arithmetic mean.

## Competing interests

The authors declare that they have no competing interests.

## Authors’ contributions

SHZ conceived of and designed the study, prepared the manuscript, and participated in data analysis. LW wrote the computer programs and participated in data analysis. All authors read and approved the final manuscript.

## Supplementary Material

Additional file 1 **List of genomes and sequences analyzed.** Excel spreadsheets containing the list of prokaryotes, eukaryotes, mitochondria, chloroplasts, plasmids, viruses and phages studied. Click here for file

Additional file 2 **Distribution of prokaryotic genomes for common class 1 and common class 2 in terms of oligonucleotide frequency profiles.** Excel spreadsheet showing the distribution of prokaryotic genomes for the two common classes. Click here for file

Additional file 3 **Details of the trinucleotide frequency profiles of prokaryotic genomes.** Excel spreadsheets showing the trinucleotide frequency profiles of prokaryotic genomes for common class 1 (common profile 1), common class 2 (common profile 2), and an example of the minority class. Click here for file

Additional file 4 **Details of the trinucleotide frequency profiles of random sequences.** Excel spreadsheets showing the trinucleotide frequency profiles of random sequences corresponding to common profile 1 or to common profile 2. Click here for file

Additional file 5 **Details of the trinucleotide frequency profiles of eukaryotic genomes.** Excel spreadsheets showing the trinucleotide frequency profiles of human, mouse, *Ashbya gossypii*, and *Ostreococcus lucimarinus* chromosomes (chromosome segments). Click here for file

Additional file 6 **Details of the trinucleotide frequency profiles of mitochondrial, chloroplast, virus and phage genomes, and plasmids.** Excel spreadsheets showing the trinucleotide frequency profiles of these genetic systems in regard to common classes. Click here for file

Additional file 7 **“Profile correlation” and “profile similarity.”** Excel spreadsheet showing the data and figures for the comparison of mononucleotide frequency profiles and that of dinucleotide frequency profiles. Click here for file
